# Kidney Transplantation in a Patient Lacking Cytosolic Phospholipase A_2_ Proves Renal Origins of Urinary PGI-M and TX-M

**DOI:** 10.1161/CIRCRESAHA.117.312144

**Published:** 2018-01-03

**Authors:** Jane A. Mitchell, Rebecca B. Knowles, Nicholas S. Kirkby, Daniel M. Reed, Matthew L. Edin, William E. White, Melissa V. Chan, Hilary Longhurst, Magdi M. Yaqoob, Ginger L. Milne, Darryl C. Zeldin, Timothy D. Warner

**Affiliations:** From the National Heart and Lung Institute, Imperial College London, United Kingdom (J.A.M., N.S.K., D.M.R.); Blizard Institute, Queen Mary University of London, United Kingdom (R.B.K., W.E.W., M.V.C., M.M.Y., T.D.W.); National Institute for Environmental Health Sciences, Research Triangle, NC (M.L.E., D.C.Z.); Department of Nephrology (W.E.W., M.M.Y.) and Immunology Department (H.L.), Barts Health NHS Trust, London, United Kingdom; and Departments of Pharmacology and Medicine, Vanderbilt University, Nashville, TN (G.L.M.).

**Keywords:** biomarkers, endothelial cells, kidney transplantation, phenotype, thromboxane A2

## Abstract

The balance between vascular prostacyclin, which is antithrombotic, and platelet thromboxane A_2_, which is prothrombotic, is fundamental to cardiovascular health. Prostacyclin and thromboxane A_2_ are formed after the concerted actions of cPLA_2_α (cytosolic phospholipase A_2_) and COX (cyclooxygenase). Urinary 2,3-dinor-6-keto-PGF_1α_ (PGI-M) and 11-dehydro-TXB_2_ (TX-M) have been taken as biomarkers of prostacyclin and thromboxane A_2_ formation within the circulation and used to explain COX biology and patient phenotypes, despite concerns that urinary PGI-M and TX-M originate in the kidney.

**Objective:**

We report data from a remarkable patient carrying an extremely rare genetic mutation in cPLA_2_α, causing almost complete loss of prostacyclin and thromboxane A_2_, who was transplanted with a normal kidney resulting in an experimental scenario of whole-body cPLA_2_α knockout, kidney-specific knockin. By studying this patient, we can determine definitively the contribution of the kidney to the productions of PGI-M and TX-M and test their validity as markers of prostacyclin and thromboxane A_2_ in the circulation.

**Methods and Results:**

Metabolites were measured using liquid chromatography-tandem mass spectrometry. Endothelial cells were grown from blood progenitors. Before kidney transplantation, the patient’s endothelial cells and platelets released negligible levels of prostacyclin (measured as 6-keto-prostaglandin F_1α_) and thromboxane A_2_ (measured as TXB_2_), respectively. Likewise, the urinary levels of PGI-M and TX-M were very low. After transplantation and the establishment of normal renal function, the levels of PGI-M and TX-M in the patient’s urine rose to within normal ranges, whereas endothelial production of prostacyclin and platelet production of thromboxane A_2_ remained negligible.

**Conclusions:**

These data show that PGI-M and TX-M can be derived exclusively from the kidney without contribution from prostacyclin made by endothelial cells or thromboxane A_2_ by platelets in the general circulation. Previous work relying on urinary metabolites of prostacyclin and thromboxane A_2_ as markers of whole-body endothelial and platelet function now requires reevaluation.

For over 40 years, the importance of balance in the production of prostanoids has been a central theme in the understanding of cardiovascular health. Attention has focused on prostacyclin derived from the vasculature, which is antithrombotic and a vasorelaxant, and thromboxane A_2_ derived from platelets, which is prothrombotic and a vasoconstrictor. Both prostacyclin and thromboxane A_2_ are formed after the concerted actions of cPLA_2_α (cytosolic phospholipase A_2_) and COX (cyclooxygenase). COX is present in 2 isoforms; COX-1 is constitutively expressed throughout the body,^1–4^ whereas COX-2 is present constitutively only in discreet regions of the body, which include the kidney.^5–8^ COX-2 is also expressed at the site of inflammation and in cancer and as such is the therapeutic target for the nonsteroidal anti-inflammatory group of drugs, which include aspirin, ibuprofen, and celecoxib.

**Editorial, see p 537**

**Meet the First Author, see p 534**

It was found early on in prostanoid research that both prostacyclin and thromboxane A_2_ are very short lived within the circulation and that measurements of either of them or their immediate metabolites were relatively uninformative. The establishment of analytic techniques to measure 2,3-dinor-6-keto-PGF_1α_ (PGI-M), a stable metabolite of prostacyclin, and 11-dehydro-TXB_2_ (TX-M), a stable metabolite of thromboxane A_2_ in urine, therefore, seemed to provide the possibility of useful biomarkers of cardiovascular health and of drug action. However, for PGI-M and TX-M to work as biomarkers, their levels in urine should reflect levels in the circulation, and although this idea has been suggested based on selective inhibition of urinary TX-M with aspirin,^9^ it has not been experimentally proven. Since this early work it has been generally assumed that PGI-M and TX-M measured in urine reflected levels in the cardiovascular system as a whole, dependent on prostacyclin production by endothelial cells and thromboxane A_2_ production by platelets.^10–15^ This assumption has now become dogma, and stable urinary metabolites of prostacyclin and thromboxane A_2_ have been used in many studies, for example, as of September 2017 an online search on PubMed with the terms urinary prostacyclin metabolite or urinary thromboxane metabolite returns over 300 and 400 papers, respectively, whereas search of clinicaltrials.gov with the term urinary prostanoid produces 48 entries. Results from these studies have apparently informed (1) drug action in clinical studies,^11^ (2) personal risk of cardiovascular disease in patient groups,^14^ and (3) a plethora of basic science relating to eicosanoids. A widely held concept derived from such studies is that prostacyclin released by endothelial cells is formed through the actions of COX-2, following from the observation that COX-2–selective drugs reduce PGI-M and relying on the assumption that PGI-M reflects the production of prostacyclin by endothelial cells.^12,15,16^ However, this idea is not universally accepted^1,2,5,6,8,16^ because conflicting observations indicate that COX-1^1–4,8,16^ is the dominant isoform within the vasculature including endothelial cells leading some of us to suggest that urinary markers of prostacyclin can be derived from the kidney^1^ where COX-2 is highly expressed.^5–7^

To date there have been no definitive models in which the renal origin of PGI-M and TX-M can be tested. However, here, we present a report of a patient with inherited human group IV A cPLA_2_α deficiency,^17^ previously found by our group to almost completely lack the vital capacity to form several eicosanoids including endothelial prostacyclin and platelet thromboxane A_2_.^17,18^ In 2015, the patient underwent a kidney transplant receiving a normal cPLA_2_ sufficient organ. The transplant has resulted in the serendipitous generation of a remarkable experimental model akin to a human whole-body cPLA_2_α knockout, kidney-specific knockin. Now, for this patient, we can determine definitively the contribution of the kidney to the production of PGI-M and TX-M and so test the relevance of these measurements as markers of prostacyclin and thromboxane A_2_ in the circulation.

## Methods

The authors declare that all supporting data are available within the article.

### Patient Details

The patient (female, of Serbian heritage, born 1966) presented at the age of 2 years with peptic ulceration, bleeding, and pyloric stenosis, which required pyloroplasty and selective vagotomy. The patient went on to have a lifelong history of gastrointestinal disease^17^ cumulating in the diagnosis of cryptogenic multifocal ulcerous stenosing enteritis.^17^ In 2014, we reported that the patient carries a homozygous 4 bp deletion (g.155574_77delGTAA) in the *PLA2G4A* gene resulting in a frameshift of 10 amino acids before a premature stop codon (p.V707fsX10) and the loss of 43 amino acids (residues 707–749) at the C terminus of group IV A cPLA_2_α. This mutation results in a complete loss of cPLA_2_α protein expression. In line with loss of cPLA_2_α, generation of eicosanoids by whole blood,^17^ isolated platelets, peripheral blood monocytes, or blood outgrowth endothelial cells obtained from the patient^18^ was dramatically reduced. Plasma and urinary levels of most eicosanoids were also accordingly much lower than the normal range in samples from the patient.^17,18^ In 2014, renal function of the patient declined because of tubulointerstitial nephritis leading to end-stage renal failure requiring dialysis during which time the patient was producing ≈1 L/d of urine. In 2015, the patient underwent a renal transplant receiving a live unrelated spousal donor kidney. After the kidney transplant had stabilized, blood and urine samples were collected for analysis using liquid chromatography-tandem mass spectrometry at 1 to 3 months post-transplant. Blood outgrowth endothelial cells were also isolated after transplant and samples collected for eicosanoid measurements after stimulation in culture. The patient received tacrolimus as antirejection therapy.

### Blood Collection and Ethics

Blood was collected by venepuncture, and urine by samples from midstream flow from healthy volunteers and the patient.

### Whole Blood Stimulation

Heparin anticoagulated whole blood was incubated with vehicle (PBS) or Horm collagen (Nycomed, St Peter, Austria). Thromboxane B_2_ levels were measured by liquid chromatography-tandem mass spectrometry in the conditioned plasma.

### Endothelial Cells

Blood outgrowth endothelial cells were grown out from progenitors in human blood as previously described.^19–22^ Once colonies emerged (between days 4 and 20), cells were expanded and maintained in Lonza EGM-2 media (Lonza, Slough, United Kingdom) +10% fetal bovine serum and experiments performed between passages 2 and 8.

Cells were plated on 48- or 96-well plates. For eicosanoid measurements, endothelial cells were primed with interleukin-1β (IL-1β; 1 ng/mL; Invitrogen, Life Technologies, Paisley, United Kingdom) to upregulate COX pathways as described previously^23^ before being treated for 30 minutes with the calcium ionophore A23187 to activate PLA_2_.

### Eicosanoid Analysis

Levels of prostanoids in urine, whole blood, and endothelial cell samples were determined by liquid chromatography-tandem mass spectrometry as previously described.^1,18,24,25^

### Statistics and Data Analysis

Data are shown as individual data points.

### Study Approval

All experiments were subject to written informed consent, local ethical approval (healthy volunteer samples for platelet/leukocyte studies; St Thomas’s Hospital Research Ethics Committee, reference 07/Q0702/24: endothelial cell studies; Royal Brompton and Harefield Hospital Research Ethics Committee, reference 08/H0708/69: patient samples; South East NHS Research Ethics Committee) and in accordance with Declaration of Helsinki principles.

## Results

Before the kidney transplant, the patient had developed end-stage kidney failure with urine production of ≈1 L/d requiring hemodialysis 3× a week. Postoperative recovery after transplant was uneventful. Her renal function normalized with blood urea nitrogen of 6.6 and creatinine of 88 μmol/L by 4 weeks post-transplant.

In healthy volunteers, PGI-M and TX-M tend to be higher in females than males.^18^ However, due to her condition, before the transplant levels of PGI-M and TX-M in the patient’s urine were low and well below levels in control donors and the published^26^ normal ranges (Figure).^17,18^ Remarkably, the new kidney restored levels of urinary PGI-M and TX-M to the normal range. This phenomenon was found to be selective to the kidney because the ability of endothelial cells from the patient to produce prostacyclin and of platelets from the patient to produce thromboxane A_2_ remained low and unchanged by the kidney transplant (Figure). Similarly, there was no increase in the levels of PGI-M within the circulation, but rather a small reduction (25±3%), when plasma samples from after transplantation (n=5) were compared with those from before transplantation (n=8).

**Figure.**
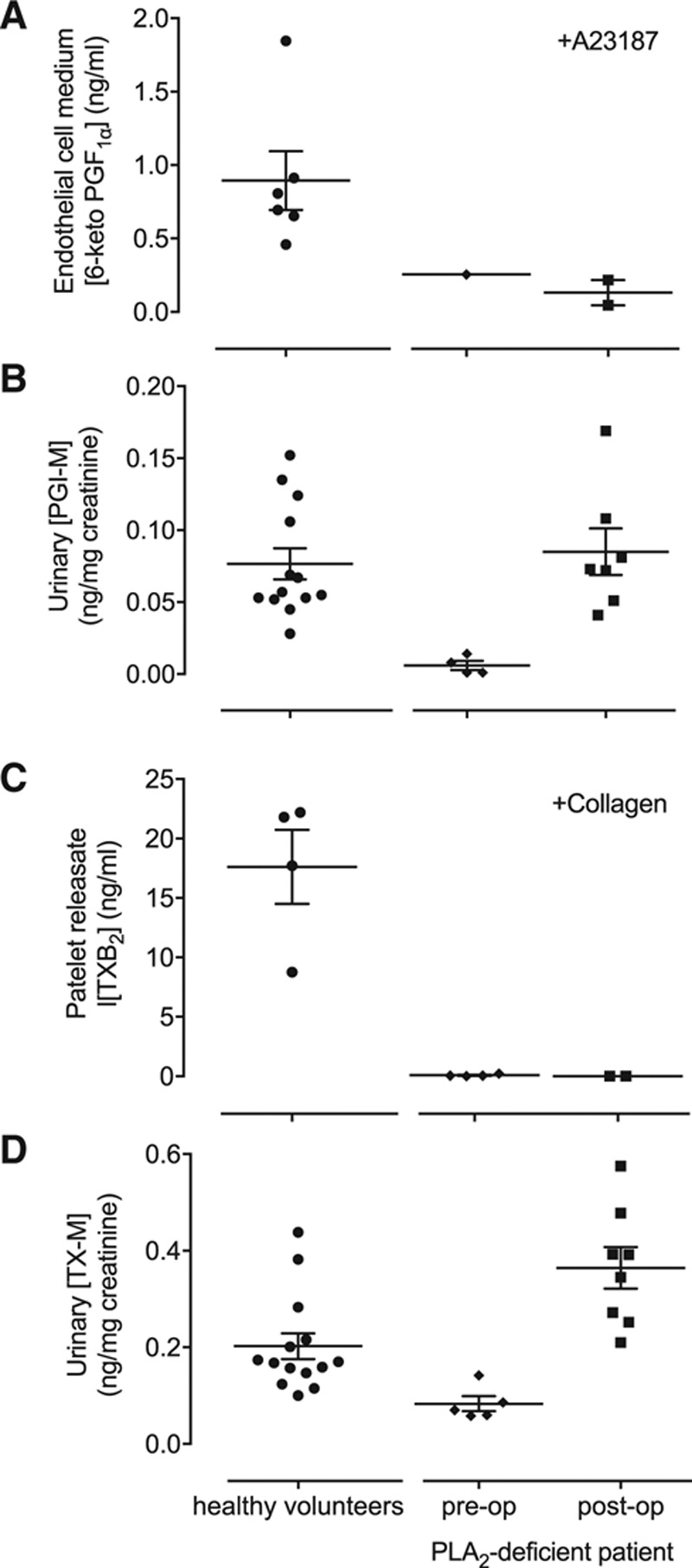
**Comparison of cellular production of eicosanoids and urinary levels of metabolites. A**, Production of prostacyclin measured as 6-keto-prostaglandin F_1α_ (6-keto-PGF_1α_) from endothelial cells stimulated with calcium ionophore (A23187); (**B**) urinary levels of the prostacyclin metabolite PGI-M (2,3-dinor-6-keto-PGF_1α_); (**C**) production of thromboxane A_2_ measured as thromboxane A_2_ (TXB_2_) from whole blood stimulated with collagen; and (**D**) urinary levels of the thromboxane metabolite TX-M (11-dehydro-TXB_2_). Measurements made in samples from healthy volunteers and from the patient before and after kidney transplantation. Results from healthy volunteers and cPLA_2_α (cytosolic phospholipase A_2_)-deficient patient pre-op includes data previously published.^18^

## Discussion

Here, we describe a remarkable clinical and experimental situation, the serendipitous generation of a unique human model in which to explore the origins of the urinary eicosanoid metabolites of prostacyclin (PGI-M) and thromboxane (TX-M).

Because eicosanoids, including prostacyclin, protect the gastrointestinal tract and the kidney, the long-term clinical symptoms of the patient can be entirely explained by the genetic deficiency and the associated lack of cPLA_2_α activity, illustrating the powerful protective role that eicosanoids play in homeostasis. After receiving a genetically normal kidney, the patient continued to be almost entirely unable to produce prostacyclin and thromboxane A_2_ from her endothelial cells and platelets. However, despite the continuing absence of endothelial prostacyclin production and platelet thromboxane A_2_ production after transplant, the patient’s urine contains apparently normal^26^ levels of PGI-M and TX-M. Importantly, it has already been demonstrated that the use of tacrolimus to reduce organ rejection in renal transplant patients is not associated with changes in either PGI-M or TX-M.^27^ It is therefore impossible in this patient that PGI-M and TX-M were derived from, or reflective of, endothelial and platelet eicosanoid productions.

These results not only describe a unique clinical case of organ transplantation in a patient with an incredibly rare gene deletion but also show unequivocally that the kidney alone can support the production of normal levels of PGI-M and TX-M and that these cannot be assumed as markers for prostacyclin and thromboxane A_2_ production within the cardiovascular system as a whole. Although there may be concerns that the patient presented here has very particular pathologies, which may not speak for normal physiological function, the same can be said for any of the many patients across a wide range of diseases in which measurement of PGI-M and TX-M has been used to describe clinical conditions.

Importantly, as mentioned above, PGI-M has been used to define the idea that endothelial cells produce prostacyclin through the action of COX-2^15^ because selective inhibitor drugs, such as celecoxib, reduce urinary PGI-M.^11^ However, this idea is not universally accepted^1,2,8,28^ and has not been supported by direct evidence, which instead identifies the ubiquitously expressed constitutive form, COX-1 as the principle driver of prostacyclin in the circulation.^1,2,28^ Similarly, there have been anomalies in the rationale that TX-M accurately reflects thromboxane A_2_ in the circulation. For example, early studies demonstrated that platelet thromboxane A_2_ production could be strongly inhibited without a concomitant reduction in urinary TX-M.^29^ At the time, this was taken as indicating the need for substantial platelet COX inhibition to reduce in vivo platelet activation. Our data now provide definitive proof for the alternative, and simpler, conclusion that both urinary PGI-M and TX-M originate from the kidney and are not necessary reflective of prostacyclin and thromboxane A_2_ in the circulation.

In conclusion, we now need to reconsider the many studies and clinical trials that have used measures of PGI-M and TX-M to construct some of the fundamental concepts of eicosanoid biology and to characterize various patient groups. This is particularly important in the areas of aspirin therapy and COX-2 biology where urinary markers have been used to inform discussions on the mechanisms associated with nonsteroidal anti-inflammatory group of drugs and cardiovascular risk.^12,15,16^ In the light of our findings, which prove that urinary PGI-M can originate from the kidney, we may conclude that earlier studies showing COX-2 inhibitor drugs to reduce PGI-M simply confirm the kidney as a prime site for constitutive COX-2 expression and add to the idea that blockade of the production of protective COX-2–derived prostanoids in the kidney contributes to nonsteroidal anti-inflammatory group of drug–induced cardiovascular side effects.

## Sources of Funding

This work was supported by the British Heart Foundation (FS/12/53/29643, FS/16/1/31699 and PG/15/47/31591), the Wellcome Trust (0852551Z108/Z), and the Intramural Research Program of the US National Institutes of Health (NIH) National Institute of Environmental Health Sciences (grant no. Z01-025034). R.B. Knowles, N.S. Kirkby, D.M. Reed, M.L. Edin, W.E. White, M.V. Chan, and G.L. Milne performed the research; J.A. Mitchell, R.B. Knowles, N.S. Kirkby, D.M. Reed, M.L. Edin, G.L. Milne, D.C. Zeldin, and T.D. Warner analyzed the data; R.B. Knowles, N.S. Kirkby, D.M. Reed, M.L. Edin, W.E. White, M.V. Chan, H. Longhurst, M.M. Yaqoob, G.L. Milne, and D.C. Zeldin edited the article; J.A. Mitchell, M.M. Yaqoob, and T.D. Warner designed the research; and J.A. Mitchell and T.D. Warner wrote the article.

## Disclosures

None.

## Novelty and Significance

### What Is Known?

- The balance of formation of the COX (cyclooxygenase)-derived eicosanoids, vascular prostacyclin, which is antithrombotic, and platelet thromboxane, A_2_, which is prothrombotic, lies at the center of cardiovascular health.
- It has been widely assumed that the production of these 2 short-lived mediators can be followed by the measurement of stable urinary metabolites, 2,3-dinor-6-keto-PGF_1α_ (PGI-M) for prostacyclin and 11-dehydro-TXB_2_ (TX-M) for thromboxane A_2_, and these measurements have been used to explain COX biology and patient phenotypes.
